# (*Z*)-Ethyl 2-oxo-3-(1,2-dihydroquinolin-2-yl­idene)propano­ate

**DOI:** 10.1107/S160053681002324X

**Published:** 2010-06-23

**Authors:** Arto Valkonen, Erkki Kolehmainen, Borys Ośmiałowski, Ryszard Gawinecki

**Affiliations:** a Department of Chemistry, University of Jyväskylä, PO Box 35, FIN-40014 Jyväskylä, Finland; b Department of Chemistry, University of Technology and Life Sciences, Seminaryjna 3, PL-85-326 Bydgoszcz, Poland

## Abstract

Both independent mol­ecules in the asymmetric unit of the tautomeric title compound, C_14_H_13_NO_3_, a synthetic product obtained from 2-lithio­methyl­quinoline and diethyl oxalate, crystallize in the enaminone form with a *Z* configuration around the double bond. Intra­molecular N—H⋯O hydrogen bonds occur, generating an *S*(6) graph-set motif. In the crystal, weak inter­molecular C—H⋯O and π–π stacking inter­actions [centroid–centroid distances = 3.7020 (14)–3.7429 (13)Å] define a three-dimensional supra­molecular network.

## Related literature

The enaminone form is predominant in the crystalline state for 2-substituted quinolines, see: Kolehmainen *et al.* (2000 ▶); Loghmani-Khouzani *et al.* (2006 ▶). The enaminone form has been found to be the only tautomeric form present in a chloro­form solution, see: More O’Ferrall & Murray (1994 ▶); Greenhill (1990 ▶). For the synthesis, see: Kolehmainen *et al.* (2000 ▶); Ośmiałowski *et al.* (2002 ▶, 2003 ▶). For its melting point, see: Stock *et al.* (1958 ▶); Leonard & Boyer (1950 ▶). For hydrogen-bond motifs, see: Bernstein *et al.* (1995 ▶). For π–π stacking inter­actions, see: Meyer *et al.* (2003 ▶). For bond-length data, see: Allen *et al.* (1987 ▶).
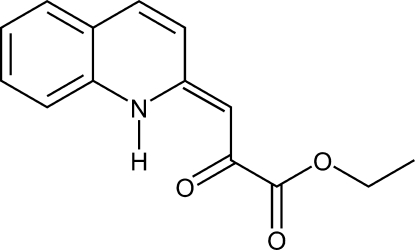


## Experimental

### 

#### Crystal data

C_14_H_13_NO_3_
*M* *_r_* = 243.25
Monoclinic, 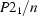

*a* = 7.8367 (3) Å
*b* = 11.9726 (6) Å
*c* = 25.3156 (13) Å
β = 99.019 (3)°
*V* = 2345.89 (19) Å^3^
*Z* = 8
Mo *K*α radiation
μ = 0.10 mm^−1^
*T* = 173 K
0.15 × 0.15 × 0.10 mm

#### Data collection

Bruker–Nonius KappaCCD diffractometer
12368 measured reflections
4210 independent reflections
2528 reflections with *I* > 2σ(*I*)
*R*_int_ = 0.084

#### Refinement

*R*[*F* ^2^ > 2σ(*F* ^2^)] = 0.050
*wR*(*F* ^2^) = 0.124
*S* = 1.01
4210 reflections
331 parameters
2 restraints
H atoms treated by a mixture of independent and constrained refinement
Δρ_max_ = 0.19 e Å^−3^
Δρ_min_ = −0.20 e Å^−3^

### 

Data collection: *COLLECT* (Bruker, 2002 ▶); cell refinement: *DENZO-SMN* (Otwinowski & Minor, 1997 ▶); data reduction: *DENZO-SMN*; program(s) used to solve structure: *SHELXS97* (Sheldrick, 2008 ▶); program(s) used to refine structure: *SHELXL97* (Sheldrick, 2008 ▶); molecular graphics: *ORTEP-3 for Windows* (Farrugia, 1997 ▶); software used to prepare material for publication: *SHELXL97* and *Mercury* (Macrae *et al.*, 2008 ▶).

## Supplementary Material

Crystal structure: contains datablocks global, I. DOI: 10.1107/S160053681002324X/jj2040sup1.cif

Structure factors: contains datablocks I. DOI: 10.1107/S160053681002324X/jj2040Isup2.hkl

Additional supplementary materials:  crystallographic information; 3D view; checkCIF report

## Figures and Tables

**Table 1:** Hydrogen-bond geometry (Å, °)

*D*—H⋯*A*	*D*—H	H⋯*A*	*D*⋯*A*	*D*—H⋯*A*
N1—H1⋯O12	0.92 (2)	1.78 (2)	2.582 (2)	144 (2)
N1*A*—H1*A*⋯O12*A*	0.91 (2)	1.87 (2)	2.633 (2)	141 (2)
C7—H7⋯O13*A*	0.95	2.50	3.203 (3)	131
C8—H8⋯O12*A*	0.95	2.51	3.456 (3)	172
C8*A*—H8*A*⋯O12	0.95	2.52	3.412 (3)	156
C16*A*—H16*D*⋯O13^i^	0.98	2.46	3.379 (3)	156

Symmetry code: (i) 
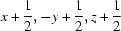
.

**Fig. 1.**
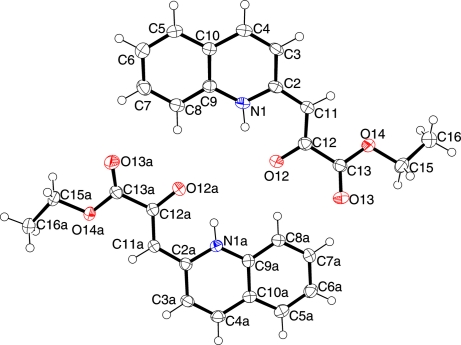
View of the asymmetric unit of title compound showing the atom-labeling scheme. Displacement ellipsoids are drawn at the 50% probability level. H atoms are represented by circles of arbitrary size.

**Fig. 2.**
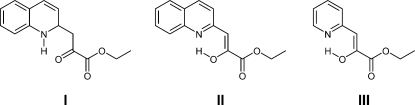
Chemical formula and structural diagrams for (Z)-Ethyl 2-oxo-3-(quinolin-2(1H)-ylidene)propanoate (I), (Z)-ethyl 2-hydroxy-3-(quinolin-2-yl)acrylate (II) and (Z)-ethyl 2-hydroxy-3-(pyridin-2-yl)acrylate (III).

**Fig. 3.**
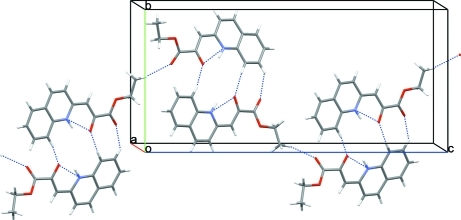
Packing diagram of the title compound showing the non-covalent N—H···O and C—H···O interactions.

## supplementary crystallographic information

### Comment

In 2-subtituted quinolines, in which the substituent contains a carbonyl group at the β-position, the enaminone form is predominant in the crystalline state, particularily at temperatures far below 0 °C (Kolehmainen *et al.*, 2000; Loghmani-Khouzani *et al.*, 2006). The title compound, (I), C_14_H_13_NO_3_, (Fig. 1) exhibits similar behavior.  Early studies were not able to determine whether the enaminone, form (I, Fig. 2) or enol,form ((*Z*)-ethyl 2-hydroxy-3-(quinolin-2-yl) acrylate (II)), was pesent in a methanol solution (Stock *et al.*, 1958). However, the enaminone form (I) has been found to be the only tautomeric form present in a chloroform solution (More O'Ferrall & Murray, 1994; Greenhill, 1990). Since the enol form, (*Z*)-ethyl 2-hydroxy-3-(pyridin-2-yl)acrylate (III), was the only tautomer detected in the chloroform solution of a pyridinyl derivative (More O'Ferrall & Murray, 1994), this suggests that benzo-annulation may be responsible for the higher stability of the enaminone form (I) over the enol form (II).

In the title compound, (I), the (*Z*)-configuration around double bond is observed in each of the two independent molecules in the asymmetric unit (Fig. 1) with only small deviations in the side chain dihedral angles. Intramolecular N—H···O hydrogen bonds (Table 1), generating an S(6) graph set motif (Bernstein *et al.*, 1995) are observed resulting from a (*Z*) configuration around C2═C11. The double-bonded O atoms are located on the same side of the C—C bond between carbonyl groups (*s*-*cis* conformation). Bond distances and angles are in normal ranges (Allen *et al.*,  1987). In addition, weak intermolecular C—H···O interactions (Table 1) as well as π–π stacking interactions with reasonable closest C···C distances (about 3.4 Å, Meyer *et al.*, 2003) are found and contribute to crystal structure stabilization.

### Experimental

(*Z*)-Ethyl 2-oxo-3-(quinolin-2(*1H*)-ylidene)propanoate was obtained from equimolar starting quantities of 2-lithiomethylquinoline and diethyl oxalate following procedures described (Kolehmainen *et al.*, 2000; Ośmiałowski *et al.*, 2002; Ośmiałowski *et al.*, 2003). The product melts at 132–134 °C (EtOH) [lit. mp 130.8–131.6 °C (Stock *et al.*, 1958); 132 °C (Leonard & Boyer, 1950)]. Suitable single crystals for X-ray diffraction were obtained by very slow evaporation of analytical sample from NMR-tube, where CDCl_3_ was used as a solvent.

### Refinement

All H atoms were visible in electron density maps, but those bonded to C were calculated at their idealized positions and allowed to ride on their parent atoms at C—H distances of 0.95 Å (aromatic), 0.98 Å (methyl) and 0.99 Å (methylene), with *U*_iso_(H) of 1.2 times *U*_eq_(C) (or 1.5 times *U*_eq_(C) for methyls). The N—H protons were found in the electron density map and were fixed in place by *DFIX* restraint (s = 0.02) at distances of 0.91 Å from N atoms, and *U*_iso_(H) values of 1.2 times *U*_eq_(N) were used.

### Crystal data

**Table:**

C_14_H_13_NO_3_	*F*(000) = 1024
*M**_r_* = 243.25	*D*_x_ = 1.378 Mg m^−^^3^
Monoclinic, *P*2_1_/*n*	Mo *K*α radiation, λ = 0.71073 Å
Hall symbol: -P 2yn	Cell parameters from 6418 reflections
*a* = 7.8367 (3) Å	θ = 0.4–26.0°
*b* = 11.9726 (6) Å	µ = 0.10 mm^−^^1^
*c* = 25.3156 (13) Å	*T* = 173 K
β = 99.019 (3)°	Block, orange
*V* = 2345.89 (19) Å^3^	0.15 × 0.15 × 0.10 mm
*Z* = 8	

### Data collection

**Table:**

Bruker–Nonius KappaCCD diffractometer	2528 reflections with *I* > 2σ(*I*)
Radiation source: fine-focus sealed tube	*R*_int_ = 0.084
graphite	θ_max_ = 25.3°, θ_min_ = 2.4°
Detector resolution: 9 pixels mm^-1^	*h* = −8→9
φ and ω scans	*k* = −14→14
12368 measured reflections	*l* = −28→30
4210 independent reflections	

### Refinement

**Table:**

Refinement on *F*^2^	Primary atom site location: structure-invariant direct methods
Least-squares matrix: full	Secondary atom site location: difference Fourier map
*R*[*F*^2^ > 2σ(*F*^2^)] = 0.050	Hydrogen site location: inferred from neighbouring sites
*wR*(*F*^2^) = 0.124	H atoms treated by a mixture of independent and constrained refinement
*S* = 1.01	*w* = 1/[σ^2^(*F*_o_^2^) + (0.0505*P*)^2^ + 0.2151*P*] where *P* = (*F*_o_^2^ + 2*F*_c_^2^)/3
4210 reflections	(Δ/σ)_max_ < 0.001
331 parameters	Δρ_max_ = 0.19 e Å^−^^3^
2 restraints	Δρ_min_ = −0.20 e Å^−^^3^

### Special details

**Table:**

Geometry. All s.u.'s (except the s.u. in the dihedral angle between two l.s. planes) are estimated using the full covariance matrix. The cell s.u.'s are taken into account individually in the estimation of s.u.'s in distances, angles and torsion angles; correlations between s.u.'s in cell parameters are only used when they are defined by crystal symmetry. An approximate (isotropic) treatment of cell s.u.'s is used for estimating s.u.'s involving l.s. planes.
Refinement. Refinement of *F*^2^ against ALL reflections. The weighted *R*-factor *wR* and goodness of fit *S* are based on *F*^2^, conventional *R*-factors *R* are based on *F*, with *F* set to zero for negative *F*^2^. The threshold expression of *F*^2^ > 2σ(*F*^2^) is used only for calculating *R*-factors(gt) *etc*. and is not relevant to the choice of reflections for refinement. *R*-factors based on *F*^2^ are statistically about twice as large as those based on *F*, and *R*- factors based on ALL data will be even larger.

### Fractional atomic coordinates and isotropic or equivalent isotropic displacement parameters (Å^2^)

**Table:**

	*x*	*y*	*z*	*U*_iso_*/*U*_eq_	
O12	0.48192 (19)	0.58816 (12)	0.21420 (6)	0.0317 (4)	
O13	0.3793 (2)	0.59651 (14)	0.10532 (7)	0.0421 (5)	
O14	0.32200 (19)	0.77845 (12)	0.11502 (6)	0.0312 (4)	
N1	0.6755 (2)	0.68740 (15)	0.29211 (8)	0.0258 (5)	
H1	0.614 (3)	0.6287 (15)	0.2746 (9)	0.031*	
C2	0.6575 (3)	0.78169 (18)	0.26243 (9)	0.0249 (5)	
C3	0.7474 (3)	0.87881 (18)	0.28434 (9)	0.0269 (5)	
H3	0.7378	0.9465	0.2645	0.032*	
C4	0.8464 (3)	0.87531 (18)	0.33324 (9)	0.0287 (6)	
H4	0.9037	0.9410	0.3476	0.034*	
C5	0.9698 (3)	0.76496 (19)	0.41398 (9)	0.0292 (6)	
H5	1.0306	0.8285	0.4296	0.035*	
C6	0.9840 (3)	0.6654 (2)	0.44071 (10)	0.0337 (6)	
H6	1.0558	0.6598	0.4745	0.040*	
C7	0.8932 (3)	0.57168 (19)	0.41838 (10)	0.0335 (6)	
H7	0.9026	0.5032	0.4376	0.040*	
C8	0.7908 (3)	0.57718 (19)	0.36916 (9)	0.0293 (6)	
H8	0.7298	0.5132	0.3542	0.035*	
C9	0.7778 (3)	0.67895 (18)	0.34137 (9)	0.0239 (5)	
C10	0.8656 (3)	0.77435 (18)	0.36343 (9)	0.0239 (5)	
C11	0.5546 (3)	0.77913 (18)	0.21150 (9)	0.0268 (6)	
H11	0.5380	0.8460	0.1911	0.032*	
C12	0.4772 (3)	0.68116 (19)	0.19047 (9)	0.0268 (5)	
C13	0.3875 (3)	0.67949 (19)	0.13238 (9)	0.0274 (6)	
C15	0.2399 (3)	0.7798 (2)	0.05944 (9)	0.0368 (6)	
H15A	0.1482	0.7223	0.0533	0.044*	
H15B	0.3260	0.7639	0.0357	0.044*	
C16	0.1634 (3)	0.8941 (2)	0.04784 (11)	0.0440 (7)	
H16A	0.1069	0.8977	0.0105	0.066*	
H16B	0.2552	0.9504	0.0540	0.066*	
H16C	0.0781	0.9089	0.0715	0.066*	
O12A	0.53371 (19)	0.35331 (12)	0.32136 (6)	0.0321 (4)	
O13A	0.7560 (2)	0.31949 (14)	0.41590 (7)	0.0503 (5)	
O14A	0.67498 (19)	0.14041 (13)	0.41249 (6)	0.0329 (4)	
N1A	0.3651 (2)	0.25757 (16)	0.23504 (7)	0.0252 (5)	
H1A	0.408 (3)	0.3175 (15)	0.2546 (8)	0.030*	
C2A	0.4166 (3)	0.15861 (18)	0.25803 (9)	0.0244 (5)	
C3A	0.3623 (3)	0.05969 (18)	0.22881 (9)	0.0280 (5)	
H3A	0.3991	−0.0111	0.2433	0.034*	
C4A	0.2594 (3)	0.06522 (19)	0.18088 (9)	0.0291 (6)	
H4A	0.2233	−0.0018	0.1623	0.035*	
C5A	0.0960 (3)	0.1818 (2)	0.10830 (9)	0.0302 (6)	
H5A	0.0552	0.1170	0.0886	0.036*	
C6A	0.0484 (3)	0.2854 (2)	0.08803 (10)	0.0325 (6)	
H6A	−0.0277	0.2920	0.0550	0.039*	
C7A	0.1120 (3)	0.38156 (19)	0.11605 (9)	0.0320 (6)	
H7A	0.0812	0.4532	0.1014	0.038*	
C8A	0.2188 (3)	0.37321 (18)	0.16452 (9)	0.0288 (6)	
H8A	0.2626	0.4385	0.1832	0.035*	
C9A	0.2620 (3)	0.26763 (18)	0.18590 (9)	0.0237 (5)	
C10A	0.2041 (3)	0.17018 (18)	0.15768 (9)	0.0242 (5)	
C11A	0.5169 (3)	0.15734 (19)	0.30907 (9)	0.0269 (5)	
H11A	0.5509	0.0873	0.3250	0.032*	
C12A	0.5685 (3)	0.25474 (18)	0.33731 (9)	0.0261 (5)	
C13A	0.6777 (3)	0.24376 (19)	0.39235 (10)	0.0292 (6)	
C15A	0.7745 (3)	0.12443 (19)	0.46547 (9)	0.0333 (6)	
H15C	0.7326	0.1753	0.4915	0.040*	
H15D	0.8981	0.1404	0.4649	0.040*	
C16A	0.7511 (3)	0.0051 (2)	0.48090 (10)	0.0416 (7)	
H16D	0.8166	−0.0088	0.5166	0.062*	
H16E	0.7933	−0.0444	0.4549	0.062*	
H16F	0.6283	−0.0096	0.4813	0.062*	

### Atomic displacement parameters (Å^2^)

**Table:**

	*U*^11^	*U*^22^	*U*^33^	*U*^12^	*U*^13^	*U*^23^
O12	0.0432 (10)	0.0213 (9)	0.0289 (10)	−0.0031 (7)	0.0007 (7)	−0.0004 (8)
O13	0.0670 (12)	0.0272 (10)	0.0301 (11)	0.0047 (8)	0.0012 (9)	−0.0086 (8)
O14	0.0386 (9)	0.0263 (9)	0.0260 (10)	0.0032 (7)	−0.0032 (7)	−0.0021 (7)
N1	0.0286 (10)	0.0194 (11)	0.0290 (12)	−0.0041 (8)	0.0038 (9)	−0.0044 (9)
C2	0.0268 (12)	0.0195 (13)	0.0299 (14)	0.0006 (9)	0.0093 (10)	−0.0025 (10)
C3	0.0329 (13)	0.0161 (12)	0.0322 (14)	−0.0017 (10)	0.0062 (10)	0.0007 (10)
C4	0.0324 (13)	0.0216 (13)	0.0326 (15)	−0.0031 (10)	0.0065 (11)	−0.0062 (11)
C5	0.0315 (13)	0.0269 (14)	0.0288 (14)	−0.0041 (10)	0.0038 (10)	−0.0068 (11)
C6	0.0376 (14)	0.0344 (15)	0.0283 (15)	−0.0009 (11)	0.0026 (11)	0.0000 (12)
C7	0.0410 (14)	0.0273 (14)	0.0329 (15)	−0.0010 (11)	0.0083 (11)	0.0051 (12)
C8	0.0325 (13)	0.0245 (13)	0.0315 (15)	−0.0015 (10)	0.0072 (10)	−0.0025 (11)
C9	0.0247 (11)	0.0244 (13)	0.0236 (13)	0.0011 (9)	0.0066 (9)	−0.0026 (11)
C10	0.0238 (11)	0.0233 (13)	0.0257 (14)	−0.0013 (9)	0.0075 (10)	−0.0021 (10)
C11	0.0321 (12)	0.0201 (13)	0.0278 (14)	0.0014 (10)	0.0032 (10)	−0.0007 (10)
C12	0.0284 (12)	0.0249 (14)	0.0278 (14)	0.0024 (10)	0.0065 (10)	−0.0018 (11)
C13	0.0315 (13)	0.0228 (13)	0.0285 (14)	0.0000 (10)	0.0063 (10)	−0.0011 (11)
C15	0.0466 (15)	0.0375 (15)	0.0237 (14)	0.0065 (12)	−0.0019 (11)	−0.0029 (12)
C16	0.0548 (17)	0.0366 (16)	0.0371 (16)	0.0038 (13)	−0.0035 (13)	0.0012 (13)
O12A	0.0420 (9)	0.0223 (9)	0.0301 (10)	0.0008 (7)	−0.0001 (7)	0.0032 (8)
O13A	0.0767 (13)	0.0298 (10)	0.0369 (12)	−0.0185 (9)	−0.0147 (10)	0.0029 (9)
O14A	0.0446 (10)	0.0260 (10)	0.0241 (10)	−0.0024 (7)	−0.0072 (7)	0.0031 (7)
N1A	0.0299 (11)	0.0231 (11)	0.0218 (11)	−0.0021 (8)	0.0019 (8)	−0.0017 (9)
C2A	0.0285 (12)	0.0220 (13)	0.0237 (13)	−0.0001 (10)	0.0080 (10)	0.0012 (10)
C3A	0.0379 (13)	0.0195 (13)	0.0274 (14)	−0.0002 (10)	0.0081 (10)	−0.0001 (11)
C4A	0.0352 (13)	0.0238 (13)	0.0287 (14)	−0.0055 (10)	0.0061 (10)	−0.0044 (11)
C5A	0.0281 (12)	0.0332 (15)	0.0285 (14)	−0.0037 (10)	0.0024 (10)	−0.0057 (11)
C6A	0.0313 (13)	0.0353 (15)	0.0285 (14)	0.0024 (11)	−0.0026 (10)	−0.0032 (12)
C7A	0.0378 (14)	0.0252 (14)	0.0314 (15)	0.0054 (11)	0.0001 (11)	0.0033 (11)
C8A	0.0333 (13)	0.0233 (13)	0.0287 (14)	0.0008 (10)	0.0015 (10)	−0.0036 (11)
C9A	0.0224 (12)	0.0252 (13)	0.0241 (13)	−0.0009 (9)	0.0056 (9)	0.0015 (10)
C10A	0.0229 (11)	0.0244 (13)	0.0260 (14)	−0.0023 (9)	0.0061 (10)	−0.0034 (10)
C11A	0.0364 (13)	0.0207 (13)	0.0232 (13)	0.0005 (10)	0.0032 (10)	0.0018 (10)
C12A	0.0308 (13)	0.0231 (13)	0.0243 (13)	−0.0001 (10)	0.0040 (10)	0.0013 (11)
C13A	0.0385 (14)	0.0225 (13)	0.0268 (14)	−0.0017 (11)	0.0054 (10)	0.0029 (11)
C15A	0.0407 (14)	0.0339 (15)	0.0217 (14)	−0.0022 (11)	−0.0062 (10)	0.0025 (11)
C16A	0.0559 (17)	0.0354 (15)	0.0291 (15)	0.0009 (12)	−0.0065 (12)	0.0053 (12)

### Geometric parameters (Å, °)

**Table:**

O12—C12	1.263 (3)	O12A—C12A	1.263 (3)
O13—C13	1.203 (3)	O13A—C13A	1.199 (3)
O14—C13	1.338 (3)	O14A—C13A	1.340 (3)
O14—C15	1.453 (3)	O14A—C15A	1.454 (2)
N1—C2	1.351 (3)	N1A—C2A	1.353 (3)
N1—C9	1.376 (3)	N1A—C9A	1.378 (3)
N1—H1	0.924 (15)	N1A—H1A	0.905 (15)
C2—C11	1.410 (3)	C2A—C11A	1.403 (3)
C2—C3	1.426 (3)	C2A—C3A	1.426 (3)
C3—C4	1.355 (3)	C3A—C4A	1.349 (3)
C3—H3	0.9500	C3A—H3A	0.9500
C4—C10	1.425 (3)	C4A—C10A	1.426 (3)
C4—H4	0.9500	C4A—H4A	0.9500
C5—C6	1.367 (3)	C5A—C6A	1.371 (3)
C5—C10	1.410 (3)	C5A—C10A	1.403 (3)
C5—H5	0.9500	C5A—H5A	0.9500
C6—C7	1.400 (3)	C6A—C7A	1.403 (3)
C6—H6	0.9500	C6A—H6A	0.9500
C7—C8	1.374 (3)	C7A—C8A	1.376 (3)
C7—H7	0.9500	C7A—H7A	0.9500
C8—C9	1.403 (3)	C8A—C9A	1.396 (3)
C8—H8	0.9500	C8A—H8A	0.9500
C9—C10	1.403 (3)	C9A—C10A	1.406 (3)
C11—C12	1.388 (3)	C11A—C12A	1.394 (3)
C11—H11	0.9500	C11A—H11A	0.9500
C12—C13	1.528 (3)	C12A—C13A	1.523 (3)
C15—C16	1.505 (3)	C15A—C16A	1.500 (3)
C15—H15A	0.9900	C15A—H15C	0.9900
C15—H15B	0.9900	C15A—H15D	0.9900
C16—H16A	0.9800	C16A—H16D	0.9800
C16—H16B	0.9800	C16A—H16E	0.9800
C16—H16C	0.9800	C16A—H16F	0.9800
			
C13—O14—C15	114.65 (17)	C13A—O14A—C15A	115.73 (17)
C2—N1—C9	124.10 (19)	C2A—N1A—C9A	123.90 (19)
C2—N1—H1	111.6 (14)	C2A—N1A—H1A	113.6 (14)
C9—N1—H1	124.3 (14)	C9A—N1A—H1A	122.5 (14)
N1—C2—C11	119.05 (19)	N1A—C2A—C11A	119.5 (2)
N1—C2—C3	117.6 (2)	N1A—C2A—C3A	117.3 (2)
C11—C2—C3	123.4 (2)	C11A—C2A—C3A	123.2 (2)
C4—C3—C2	120.5 (2)	C4A—C3A—C2A	120.9 (2)
C4—C3—H3	119.8	C4A—C3A—H3A	119.5
C2—C3—H3	119.8	C2A—C3A—H3A	119.5
C3—C4—C10	120.9 (2)	C3A—C4A—C10A	120.9 (2)
C3—C4—H4	119.6	C3A—C4A—H4A	119.5
C10—C4—H4	119.6	C10A—C4A—H4A	119.5
C6—C5—C10	120.6 (2)	C6A—C5A—C10A	121.0 (2)
C6—C5—H5	119.7	C6A—C5A—H5A	119.5
C10—C5—H5	119.7	C10A—C5A—H5A	119.5
C5—C6—C7	120.2 (2)	C5A—C6A—C7A	119.9 (2)
C5—C6—H6	119.9	C5A—C6A—H6A	120.0
C7—C6—H6	119.9	C7A—C6A—H6A	120.0
C8—C7—C6	121.0 (2)	C8A—C7A—C6A	120.7 (2)
C8—C7—H7	119.5	C8A—C7A—H7A	119.7
C6—C7—H7	119.5	C6A—C7A—H7A	119.7
C7—C8—C9	118.9 (2)	C7A—C8A—C9A	119.2 (2)
C7—C8—H8	120.6	C7A—C8A—H8A	120.4
C9—C8—H8	120.6	C9A—C8A—H8A	120.4
N1—C9—C8	120.3 (2)	N1A—C9A—C8A	120.1 (2)
N1—C9—C10	118.7 (2)	N1A—C9A—C10A	118.89 (19)
C8—C9—C10	121.0 (2)	C8A—C9A—C10A	121.0 (2)
C9—C10—C5	118.3 (2)	C5A—C10A—C9A	118.2 (2)
C9—C10—C4	118.2 (2)	C5A—C10A—C4A	123.8 (2)
C5—C10—C4	123.5 (2)	C9A—C10A—C4A	117.98 (19)
C12—C11—C2	121.5 (2)	C12A—C11A—C2A	122.6 (2)
C12—C11—H11	119.3	C12A—C11A—H11A	118.7
C2—C11—H11	119.3	C2A—C11A—H11A	118.7
O12—C12—C11	125.8 (2)	O12A—C12A—C11A	125.9 (2)
O12—C12—C13	114.90 (19)	O12A—C12A—C13A	115.81 (19)
C11—C12—C13	119.2 (2)	C11A—C12A—C13A	118.2 (2)
O13—C13—O14	124.1 (2)	O13A—C13A—O14A	123.1 (2)
O13—C13—C12	122.3 (2)	O13A—C13A—C12A	124.1 (2)
O14—C13—C12	113.55 (19)	O14A—C13A—C12A	112.81 (18)
O14—C15—C16	107.56 (19)	O14A—C15A—C16A	107.21 (18)
O14—C15—H15A	110.2	O14A—C15A—H15C	110.3
C16—C15—H15A	110.2	C16A—C15A—H15C	110.3
O14—C15—H15B	110.2	O14A—C15A—H15D	110.3
C16—C15—H15B	110.2	C16A—C15A—H15D	110.3
H15A—C15—H15B	108.5	H15C—C15A—H15D	108.5
C15—C16—H16A	109.5	C15A—C16A—H16D	109.5
C15—C16—H16B	109.5	C15A—C16A—H16E	109.5
H16A—C16—H16B	109.5	H16D—C16A—H16E	109.5
C15—C16—H16C	109.5	C15A—C16A—H16F	109.5
H16A—C16—H16C	109.5	H16D—C16A—H16F	109.5
H16B—C16—H16C	109.5	H16E—C16A—H16F	109.5
			
C9—N1—C2—C11	177.4 (2)	C9A—N1A—C2A—C11A	−177.9 (2)
C9—N1—C2—C3	−1.5 (3)	C9A—N1A—C2A—C3A	1.0 (3)
N1—C2—C3—C4	−0.2 (3)	N1A—C2A—C3A—C4A	−1.9 (3)
C11—C2—C3—C4	−179.0 (2)	C11A—C2A—C3A—C4A	177.0 (2)
C2—C3—C4—C10	1.2 (4)	C2A—C3A—C4A—C10A	1.0 (3)
C10—C5—C6—C7	−0.8 (4)	C10A—C5A—C6A—C7A	1.9 (4)
C5—C6—C7—C8	1.0 (4)	C5A—C6A—C7A—C8A	−1.6 (4)
C6—C7—C8—C9	−0.2 (4)	C6A—C7A—C8A—C9A	−0.6 (4)
C2—N1—C9—C8	−179.1 (2)	C2A—N1A—C9A—C8A	−178.3 (2)
C2—N1—C9—C10	2.0 (3)	C2A—N1A—C9A—C10A	0.8 (3)
C7—C8—C9—N1	−179.7 (2)	C7A—C8A—C9A—N1A	−178.3 (2)
C7—C8—C9—C10	−0.8 (3)	C7A—C8A—C9A—C10A	2.7 (3)
N1—C9—C10—C5	179.9 (2)	C6A—C5A—C10A—C9A	0.1 (3)
C8—C9—C10—C5	1.0 (3)	C6A—C5A—C10A—C4A	−179.6 (2)
N1—C9—C10—C4	−0.9 (3)	N1A—C9A—C10A—C5A	178.5 (2)
C8—C9—C10—C4	−179.7 (2)	C8A—C9A—C10A—C5A	−2.4 (3)
C6—C5—C10—C9	−0.2 (3)	N1A—C9A—C10A—C4A	−1.7 (3)
C6—C5—C10—C4	−179.4 (2)	C8A—C9A—C10A—C4A	177.3 (2)
C3—C4—C10—C9	−0.7 (3)	C3A—C4A—C10A—C5A	−179.4 (2)
C3—C4—C10—C5	178.6 (2)	C3A—C4A—C10A—C9A	0.9 (3)
N1—C2—C11—C12	−2.5 (3)	N1A—C2A—C11A—C12A	−0.9 (3)
C3—C2—C11—C12	176.3 (2)	C3A—C2A—C11A—C12A	−179.7 (2)
C2—C11—C12—O12	3.6 (4)	C2A—C11A—C12A—O12A	−0.2 (4)
C2—C11—C12—C13	−171.9 (2)	C2A—C11A—C12A—C13A	−179.8 (2)
C15—O14—C13—O13	−0.9 (3)	C15A—O14A—C13A—O13A	−0.2 (3)
C15—O14—C13—C12	178.48 (19)	C15A—O14A—C13A—C12A	−179.0 (2)
O12—C12—C13—O13	−26.2 (3)	O12A—C12A—C13A—O13A	−14.1 (4)
C11—C12—C13—O13	149.7 (2)	C11A—C12A—C13A—O13A	165.5 (2)
O12—C12—C13—O14	154.39 (19)	O12A—C12A—C13A—O14A	164.6 (2)
C11—C12—C13—O14	−29.7 (3)	C11A—C12A—C13A—O14A	−15.7 (3)
C13—O14—C15—C16	176.16 (19)	C13A—O14A—C15A—C16A	178.8 (2)

### Hydrogen-bond geometry (Å, °)

**Table:**

*D*—H···*A*	*D*—H	H···*A*	*D*···*A*	*D*—H···*A*
N1—H1···O12	0.92 (2)	1.78 (2)	2.582 (2)	144 (2)
N1A—H1A···O12A	0.91 (2)	1.87 (2)	2.633 (2)	141 (2)
C7—H7···O13A	0.95	2.50	3.203 (3)	131
C8—H8···O12A	0.95	2.51	3.456 (3)	172
C8A—H8A···O12	0.95	2.52	3.412 (3)	156
C16A—H16D···O13^i^	0.98	2.46	3.379 (3)	156

Symmetry codes: (i) *x*+1/2, −*y*+1/2, *z*+1/2.
